# Metabolic flux analysis for metabolome data validation of naturally xylose-fermenting yeasts

**DOI:** 10.1186/s12896-019-0548-0

**Published:** 2019-08-05

**Authors:** Henrique C. T. Veras, Christiane G. Campos, Igor F. Nascimento, Patrícia V. Abdelnur, João R. M. Almeida, Nádia S. Parachin

**Affiliations:** 1 Grupo Engenharia de Biocatalisadores, Universidade de Brasília - UnB , Campus Darcy Ribeiro, Instituto de Ciências Biológicas, Bloco K, 1° andar, Asa Norte, Brasilia, 70.790-900 Brazil; 2 Empresa Brasileira de Pesquisa Agropecuária, EMBRAPA Agroenergia, Brasília-DF, Brazil; 3 Instituto de Química, Universidade Federal de Goiás - UFG, Goiânia, Brazil; 4 Programa de Pós-Graduação em Biologia Microbiana, Instituto de Biologia, Universidade de Brasília - UnB, Brasilia, Brazil; 5 Programa de Pós-Graduação em Administração, Universidade de Brasília - UnB, Brasília, Brazil

**Keywords:** Xylose metabolism, MFA, Metabolome, Ethanol, Cofactor balance, Metabolomics

## Abstract

**Background:**

Efficient xylose fermentation still demands knowledge regarding xylose catabolism. In this study, metabolic flux analysis (MFA) and metabolomics were used to improve our understanding of xylose metabolism. Thus, a stoichiometric model was constructed to simulate the intracellular carbon flux and used to validate the metabolome data collected within xylose catabolic pathways of non-*Saccharomyces* xylose utilizing yeasts.

**Results:**

A metabolic flux model was constructed using xylose fermentation data from yeasts *Scheffersomyces stipitis*, *Spathaspora arborariae*, and *Spathaspora passalidarum*. In total, 39 intracellular metabolic reactions rates were utilized validating the measurements of 11 intracellular metabolites, acquired by mass spectrometry. Among them, 80% of total metabolites were confirmed with a correlation above 90% when compared to the stoichiometric model. Among the intracellular metabolites, fructose-6-phosphate, glucose-6-phosphate, ribulose-5-phosphate, and malate are validated in the three studied yeasts. However, the metabolites phosphoenolpyruvate and pyruvate could not be confirmed in any yeast. Finally, the three yeasts had the metabolic fluxes from xylose to ethanol compared. Xylose catabolism occurs at twice-higher flux rates in *S. stipitis* than *S. passalidarum* and *S. arborariae*. Besides, *S. passalidarum* present 1.5 times high flux rate in the xylose reductase reaction NADH-dependent than other two yeasts.

**Conclusions:**

This study demonstrated a novel strategy for metabolome data validation and brought insights about naturally xylose-fermenting yeasts. *S. stipitis* and *S. passalidarum* showed respectively three and twice higher flux rates of XR with NADH cofactor, reducing the xylitol production when compared to *S. arborariae*. Besides then, the higher flux rates directed to pentose phosphate pathway (PPP) and glycolysis pathways resulted in better ethanol production in *S. stipitis* and *S. passalidarum* when compared to *S. arborariae*.

**Electronic supplementary material:**

The online version of this article (10.1186/s12896-019-0548-0) contains supplementary material, which is available to authorized users.

## Background

Several non-*Saccharomyces* yeasts capable of naturally utilize xylose as carbon source have been identified [1–7]. Among them, *Scheffersomyces stipitis* is one of the most studied species and the *Spathaspora ssp*. has attracted attention in recent years [8–10]. The interest in the economic conversion of this pentose sugar, present in lignocellulosic biomass, to fuels and chemicals, motivated the study of xylose consumption in novel yeasts [4, 11–13]. However, fully understand the xylose metabolism is still a challenge to improve the use of this sugar, the second more abundant in nature, as a carbon source [14]. Therefore, the systems biology approach will be useful for the identification of principles and patterns that characterize the metabolism of xylose.

Metabolic flux analysis (MFA) is used to estimate the intracellular fluxes under a defined metabolic network [15, 16]. It gives insights on how metabolism is balanced, that is, how organisms convert substrates into biomass and chemicals products [1, 17]. Thus, MFA is useful for the prediction of possible metabolic limitations. It can contribute to strain engineering towards high yields of lignocellulosic ethanol production [18–20]. The metabolic networks constructed for MFA, commonly, use the information available from genome annotation. A set of enzymatic reactions are identified and converted into a mathematical model [21]. Several bioinformatics tools are available to perform MFA. Among them, OptFlux is an open-source platform that allows in silico simulations of intracellular carbon fluxes distribution into a defined metabolic network [22]. The constraint-based flux analysis, included in the OptFlux platform, establishes a set of measured extracellular fluxes such as substrate uptake and products formation rates to determine the carbon flux distribution [15]. The number of measured fluxes determines the size of the network. Therefore, a higher number of measured fluxes results in a more accurate metabolic network.

The understating of a real state of a cell depends on a set of analyzes that provides dataset about the genome, transcriptome, proteome, and metabolome [21]. Among those, the metabolome dataset is advantageous since quantification of intracellular metabolites can be directly linked to the metabolic network reflecting the phenotype of an organism at that moment [23, 24]. The systems biology approach, considering the combination of two methods such as MFA and metabolomics, is a valuable strategy to predict intracellular metabolic fluxes distribution and to understand the behavior of a given metabolic network.

Nevertheless, the utilization of data from intracellular metabolite quantification is not routinely incorporated into MFA due to various technical challenges [18, 25]. Among them, there are the steps of data acquisition, such as sample preparation and metabolites extraction [26, 27], which are critical because of the high turnover rates of intracellular reactions [28]. Then, it needs the establishment of a sensitive and selective analytical method for detection and quantification the metabolites taking into consideration the low concentration of metabolites in a complex biological matrix [26, 28]. Finally, the amount of data generated demands statistical analysis so a trustable value can be applied to MFA [29, 30].

Thereby, the purpose of this work was to validate a dataset of 11 intracellular metabolites of naturally xylose-fermenting yeasts utilizing MFA. Thus, for the first time, a comparative evaluation of metabolic flux analysis with addition metabolome data was performed for *Scheffersomyces stipitis, Spathaspora passalidarum,* and *Spathaspora arborariae.*

Among them, 80% of total metabolites were confirmed with a correlation above 90% when compared to the stoichiometric model. Nevertheless, the metabolites phosphoenolpyruvate and pyruvate could not be validated in any studied yeasts. Finally, the three yeasts had the metabolic fluxes from xylose to ethanol compared. Xylose catabolism occurs at twice-higher flux rates in *S. stipitis* than *S. passalidarum* and *S. arborariae*. In yeasts *S. stipitis* and *S. passalidarum* is observed that after the xylose assimilation reactions, approximately 50% of the carbon flux rates are directed to pentose phosphate pathway (PPP) and 50% to glycolysis. Different from *S. arborariae*, where first, carbon flux is directed to reaction into oxidative-PPP. Besides, *S. passalidarum* present 1.5 times high flux rate in the xylose reductase reaction NADH-dependent than other two yeasts.

## Results

### MFA for xylose-fermenting yeasts *S. stipitis*, *S. arborariae,* and *S. passalidarum*

Initially, one stoichiometric model was constructed for *S. stipitis*, *S. arborariae,* and *S. passalidarum* containing the xylose catabolism, pentose phosphate pathway, glycolysis, and tricarboxylic acid cycle. The model has 39 reactions and 35 metabolites, including the cofactors NAD(P) H, NAD(P)^+^, and ATP (Additional files 1, 2 and 3). The difference between the number of reactions and metabolites resulted in four degrees of freedom. Table 1 shows the measured extracellular rates included in the model. Different time points were used to calculate the rates because of the different growth rates between the yeasts. Therefore all samples were taken at the exponential phase at 28 h, 32 h, and 40 h for *S. stipitis*, S*. arborariae*, and *S. passalidarum*, respectively (Table 1).

**Table 1:**
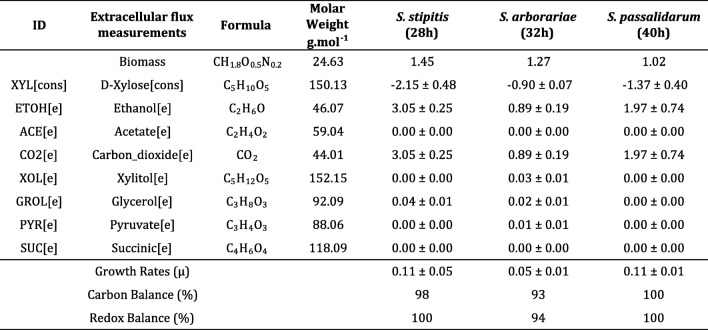
Measured extracellular flux rates

Xylose consumption rates [cons] are represent by a negative signal, extracellular product formation [e] (mmol/gCDW.h^− 1^), Carbon balance (%), and redox balance (%). The experiments were performed with sample withdraw at the exponential growth phase in biological triplicates

**Fig. 1:**
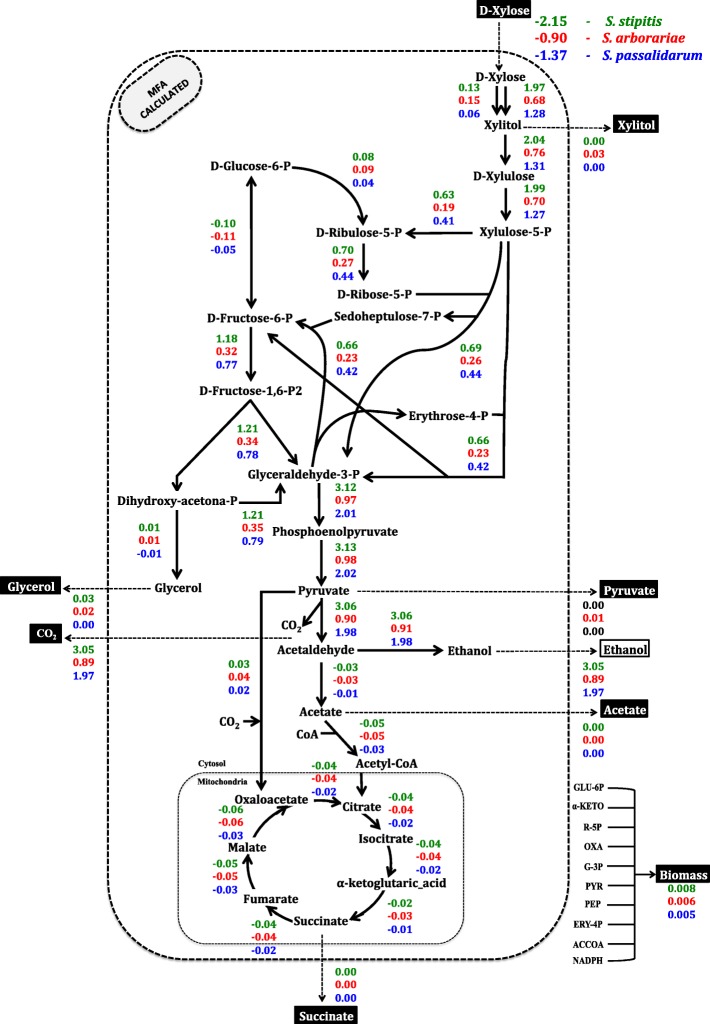
Intracellular carbon fluxes distributions during xylose catabolism to ethanol production. *S. stipitis* (green)*, S. arborariae* (red), *and S. passalidarum* (blue). Xylose consumption rates are represented by a negative signal. The first intracellular reaction (xylose to xylitol) shows two arrows; left represents reaction using NADPH, right represents reaction using NADH cofactor. The extracellular metabolites highlighted in black boxes had its flux rates used as constraints to the MFA-calculated

The rates of extracellular metabolite were used as constraints to simulate the intracellular carbon flux distributions in the MFA model (Fig. 1). The xylose consumption rates of respectives yeasts are represents by a negative signals. The extracellular xylose consumption rate of *S. stipitis* is at least twice faster than observed in *S. arborariae*, while the *S. passalidarum* showed 1.5 times faster xylose consumption rate than *S. arborariae.*

**Fig. 2:**
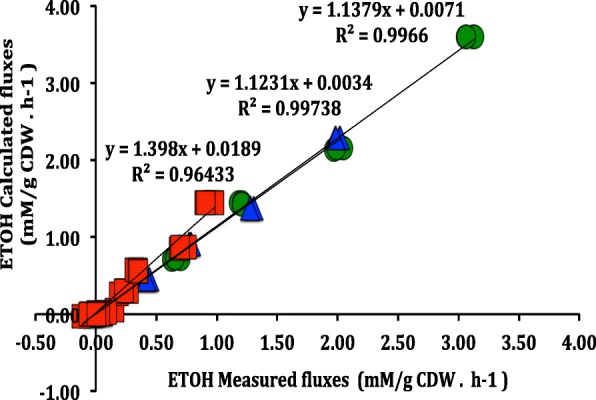
Correlation between measured and calculated fluxes for ethanol production. *S. stipitis* (green cycle), *S. arborariae* (red square) and *S. passalidarum* (blue triangle)

Among product formation, ethanol was the major metabolite secreted by all the three yeasts evaluated. Therefore, the respective ethanol measurements rates were used to validate the intracellular flux simulations. The correlation between experimentally and calculated rates for ethanol production were above 90% (Fig. 2). Accordingly, we defined the respective intracellular carbon flux distributions as a metabolic flux calculated (MFA-calculated). In other words, the MFA-calculated is the one that the intracellular carbon flux distribution was simulated utilizing the extracellular rates.

In our analysis of MFA, we can identify that some metabolites influence the flux distribution. For example, the reactions for conversion the metabolites erythrose-4-phosphate and D-Ribulose-5-phosphate are 2.8 and 1.8 times lower in *S. arborariae* (R09 = 0.23) than observed in *S. stipitis* (R09 = 0.66) and *S. passalidarum* (R09 = 0.42), respectively. Also, it was found that ethanol production rates are inversely proportional to glycerol and xylitol metabolites formation.

### Metabolomics of *S. stipitis, S. arborariae,* and *S. passalidarum* during growth on xylose as a carbon source

**Fig. 3:**
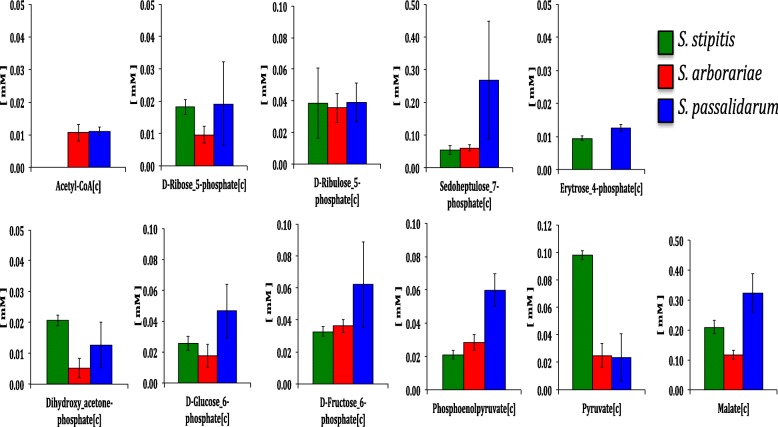
Intracellular metabolites concentrations (mM). Yeast *S. stipitis* (green column), *S. arborariae* (red column), and *S. passalidarum* (blue column). All experiments performed in biological triplicates. [c] represent the cytosol metabolites. The presented values are the average of ANOVA analysis for three biological replicates and nine technical replicates

The quantification of the 11 metabolites concentration (mM) in all studied yeasts is shown in Fig. 3. The analysis of variance (ANOVA) could corroborate that the metabolome data are within a range of reliability (Additional file 4). The metabolite ACCOA could not be detected only in *S. stipitis* but was detected in *S. arborariae* and *S. passalidarum*, indicating that in *S. stipitis* less carbon was directed into respiratory metabolism. The RU5P (0.04 mM) is twice concentrated than R5P (0.02 mM) in both *S. stipitis* and *S. passalidarum*. While in *S. arborariae,* the R5P concentration (0.01 mM) was three times lower than RU5P (0.04 mM). The concentration of S7P is seven times higher in *S. passalidarum* than observed in *S. stipitis* and *S. arborariae*. The metabolite E4P was not detected only *S arborariae* but was detected in *S. stipitis* and *S. passalidarum*, indicating that E4P may rapidly be converted to G3P and F6P. The DHAP was about four times lower in *S. arborariae* (0.005 mM) than observed in *S. stipitis* (0.020 mM) and *S. passalidarum* (0.015 mM). The metabolites G6P (0.05 mM), F6P (0.06 mM) and PEP (0.06 mM) were at least twice concentrated in *S. passalidarum* than observed in *S. stipitis* (0.02 mM, 0.03 mM and 0.02 mM) and *S. arborariae* (0.02 mM, 0.03 mM and 0.02 mM), respectively. The metabolite PYR in *S. stipitis* (0.10 mM) is five times higher than observed in *S. arborariae* (0.02 mM) and *S. passalidarum* (0.02 mM). Finally, the concentration of MAL in *S. arborariae* (0.10 mM) was twice and three times lower than observed in *S. stipitis* (0.20 mM) and *S. passalidarum* (0.30 mM), respectively (Fig. 3).

### Utilization of the NAD(P) H and NAD(P)^+^ cofactors along with the metabolic network

**Fig. 4:**
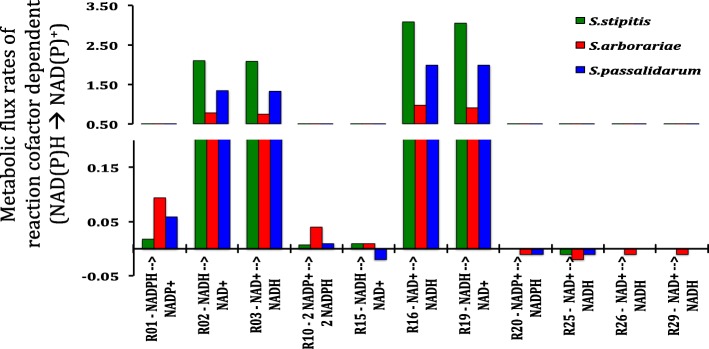
Metabolic reaction rates using NAD(P) H / NAD(P)^+^ cofactors. *S. stipitis* (green), *S. arborariae* (red), and *S. passalidarum* (blue). R01 – XYL to XOL; R02 - XYL to XOL; R03 – XOL to XYLU; R10 – G6P to RU5P; R15 – DHAP to GOL; R16 – GAP to PEP; R19 – ACCOA to ETOH; R20 – ACDH to ACE; R25 – ISO to AKG; R26 – AKG to SUC; and R29 – MAL to OXA

The metabolic model constructed contains specific reaction rates cofactors NAD(P) H – NAD(P)^+^ dependent. The NADPH/NADP^+^ ratio is twice higher in *S. stipitis*, while in *S. arborariae* and *S. passalidarum* we did not observe differences. The high NADPH released can influence the higher biomass formation observed in *S. stipitis* in contrast with *S. arborariae* and *S. passalidarum*. Already the NAD^+^/NADH ratio is 2.5 times high in *S. arborariae*, showing high NAD^+^ released, which characterize an unbalance cofactor in this yeast. Among the simulations of intracellular carbon flux distribution, some reaction rates caught our attention (Fig. 4). For example, the reaction rate (R02) that converts XYL **→** XOL with NADH-dependent was three times higher than the reaction rate using NADPH. The reaction rate G6P + 2NADP **→** RU5P + 2NADPH (R10) in *S. arborariae* was four times higher than observed in *S. stipitis* and *S. passalidarum.* Showing that in the yeast *S. arborariae,* there is a higher necessity of regeneration the NADPH cofactor. Corroborating the high reaction rate (R01) that use NADPH in reaction conversion. Besides, the yeast *S. passalidarum* showed the reaction rate DHAP + NADH **→** GROL + NAD^+^ (R15) negative, contrasting with *S. stipitis* and *S. arborariae*. This characteristic in *S. passalidarum* agrees with the absence of glycerol formation and better cofactors balance. The reactions XYL + NADH **→** XOL + NAD^+^ (R02), XOL + NAD^+^ **→** XYLO + NADH (R03), GAP + NAD^+^ **→** PEP + NADH (R16), and ACDH + NADH **→** ETOH + NAD^+^ (R19) presented the highest rates. Indicating the importance of cofactor balance. The reaction rates with negative values indicate reversible reactions.

### Validation of metabolome dataset utilizing MFA

**Fig. 5:**
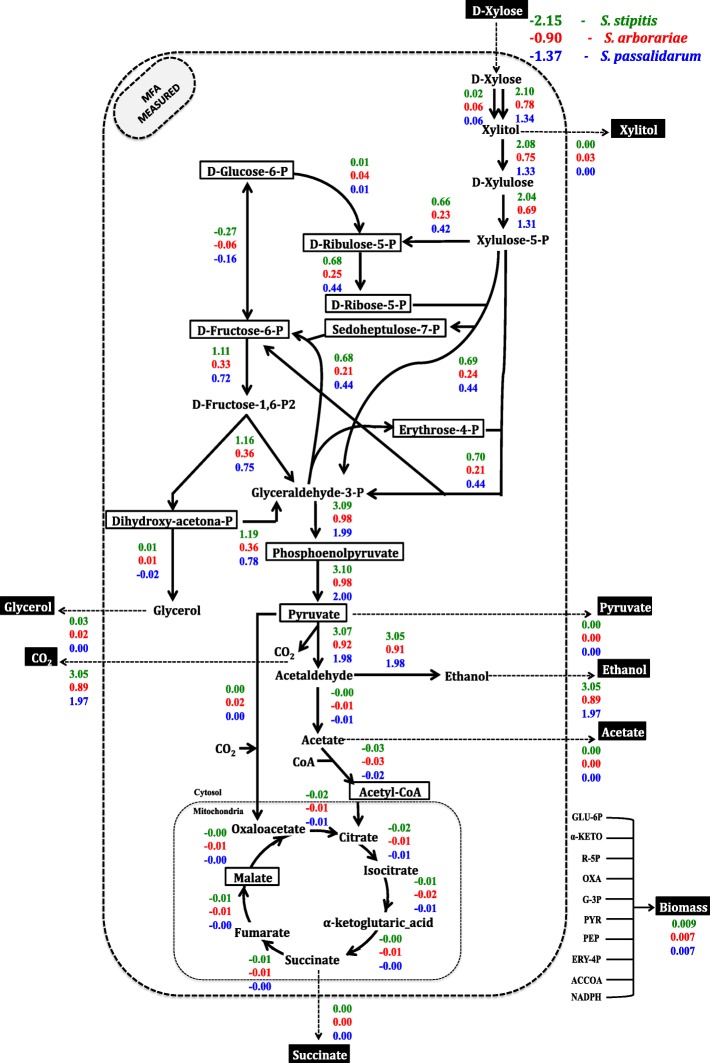
Intracellular carbon flux distribution using measured data. *S. stipitis* (green)*, S. arborariae* (red), *and S. passalidarum* (blue). Xylose consumption rates are represented by a negative signal. The first reaction (xylose to xylitol) shows two arrows; left represents reaction using NADPH, right represents reaction using NADH cofactor. Extracellular metabolites highlighted in black boxes had the flux rates used as constraints. Intracellular metabolites highlighted in white boxes limited the reaction rates with its concentration in MFA-measured

The metabolite quantification was validated using MFA analysis. For that, the entire metabolome dataset was added to the MFA-calculated (Fig. 5). The metabolic flux model with metabolome data is defined as an MFA-measured. First, one metabolite was added per simulation and compared to the fluxes obtained experimentally in the MFA-Calculated. In *S. stipitis* and *S. arborariae*, ten intracellular metabolites were quantified and, consequently, ten simulations performed. While in *S. passalidarum*, 11 metabolites quantified, which resulted in 11 simulations of carbon flux distribution.

In the yeast *S. stipitis,* it was observed that from ten metabolites, eight (i.e., 80%) showed a correlation higher than 0.90 between calculated and measured fluxes (Additional file 5a). Only in *S. stipitis,* there was no simulation for metabolic flux distribution measured with ACCOA since it could not be detected experimentally. In its turn, in *S. arborariae,* it was observed that from ten metabolites, seven (i.e., 70%) showed a correlation higher than 0.90 between calculated and measured fluxes (Additional file 5b). Only on *S. arborariae,* no simulation occurred with metabolite E4P since it could not be detected experimentally. While in *S. passalidarum,* it was observed that from 11 metabolites, nine (i.e., 82%) showed a correlation higher than 0.90 between calculated and measured fluxes (Additional file 5c).

**Fig. 6:**
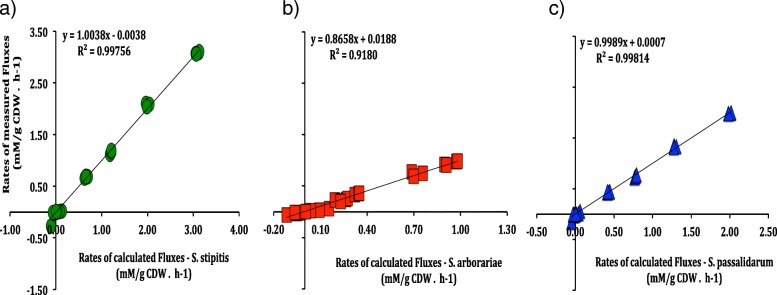
Correlation test (R^2^) between calculated and measured flux rates (mM/gCDW.h^− 1^). The relationship assessed with all metabolites measured that present a correlation higher than 0.90 for *S. stipitis* (**a**)*, S. arborariae* (**b**), and *S. passalidarum.* (**c**)

The consistency of intracellular metabolites measurements were verified using the Pearson correlation test (R^2^) (Fig. 6). The correlation encountered between experimental data and calculated flux obtained for *S. stipitis*, *S. arborariae,* and *S. passalidarum* were above 90%*.* Overall, from 20 metabolic flux rates involved in xylose conversion to ethanol, only four of them were not predicted accurately in the metabolic model proposed. Therefore the metabolic flux distribution measured compared with metabolic flux calculated ones confirmed the accuracy of the metabolome data. Although most of the metabolites had a correlation above 90%, it is noted that the measured and calculated metabolic fluxes for the metabolites PEP (29%) and PYR (69%) has a weak correlation in three tested xylose-fermenting yeasts.

**Fig. 7:**
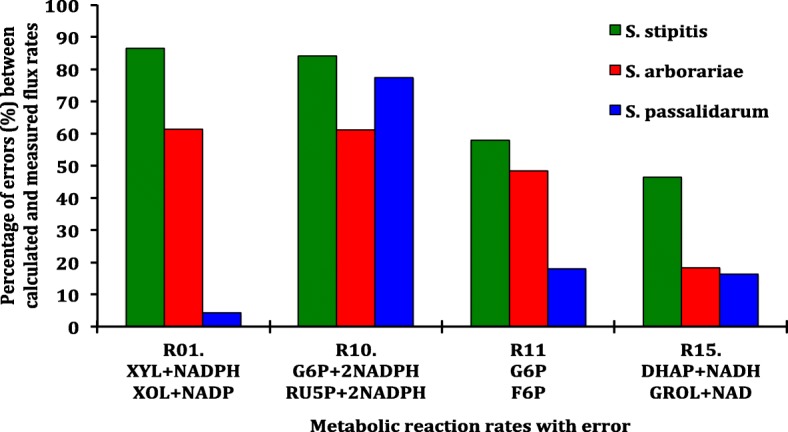
Percentage of errors between calculated and measured flux rates. Columns are showing that the errors are less than 10% for most of the metabolic reaction rates from xylose to ethanol: *S. stipitis* (green), *S. arborariae* (red), and *S. passalidarum* (blue)

The flux rates of *S. stipitis* and *S. arborariae* in the xylose assimilation pathway showed that the reaction XYL + NADPH **→** XOL + NADP (R01) have an error of 85 and 60%, respectively (Fig. 7). Figure 7 highlighted only the reaction rates that errors were above 10%. The reaction rate using cofactor NADPH influenced the xylitol accumulation and interfered the flux analysis.

The reaction rate G6P **→** RU5P (R10), responsible for regenerating NADPH in oxidative pentose phosphate pathway and the reversible reaction G6P **← →** F6P, also showed error higher than 60%. In contrast, in *S. passalidarum,* the reaction rate G6P **→** F6P (R11) showed at least 2.5 times less error when compared to *S. stipitis* and *S. arborariae*. In general, our analysis was able to predict 80% of intracellular carbon flux rates with an accuracy above 90% in relationship to calculated and measured flux rates from xylose until ethanol formation.

## Discussion

### Metabolome dataset for the intracellular carbon flux distribution

Metabolome data increase the precision of the actual state of cell metabolism [21]. Its measurements can be directly linked to the metabolic network since it enables the identification and quantification a large number of metabolites simultaneously under a specific condition [24, 29]. The metabolomic analyses result in the generation of a complex dataset. Some of the technical challenges are processing a large amount of data and performing statistical analyses, and then it can be linked to the studied biological system [30]. Therefore, it is crucial to develop an approach that is capable of validating metabolome data after statistical treatment.

Previous metabolomic studies did not succeed in quantifying the sugar-phosphate as such as G6P and F6P [31, 32]*.* Nevertheless, here, both isomers could be detected and quantified in the three yeasts *S. stipitis*, *S. arborariae*, and *S. passalidarum*. Also, the method based on UHPLC-MS/MS employed in this study enabled the separation and quantification of RU5P and R5P [26], overcoming the limitation observed in a previous metabolomics analysis for xylose fermentation performed by *S. passalidarum* [33].

Some metabolites could not be detected in particular yeasts; for example, E4P could not be detected only in *S. arborariae*. Since the flux rate formation for E4P in this yeast is at least twice slowly (reaction S7P + GAP **→** E4P + F6P = 0.23) when compared to *S. stipitis* and *S. passalidarum* (reaction S7P + GAP **→** E4P + F6P = 0.65 and 0.42). Therefore this metabolite was below the detection limit. Also, the absence of E4P is associated with low carbon flux rate into the pentose phosphate pathway in *S. arborariae*, when compared to *S. stipitis* and *S. passalidarum*. The ACCOA could not be detected in *S. stipitis* since it is observed that the carbon flux is preferably directed to ethanol formation.

Consequently, less carbon flux is directed to respiratory metabolism in the tricarboxylic acid cycle. Also, the difficulty of accurately quantifying metabolic flux influenced the detection of the ACCOA metabolite. Possibly, this limitation can be solved with the addition of more metabolic reactions in the stoichiometric model. Moreover, the ACCOA is involved in 34 compartmentalized metabolic reactions and used for acetylation of macromolecules [34]. To cell balance the use of this precursor metabolite, cells have evolved several levels of tight regulation, especially to control the biosynthesis of amino acids, lipids, nucleotides, and carbohydrates needed for cell growth, homeostasis, and maintenance [34].

The metabolites PEP and PYR could not be validated using MFA in any studied yeast. However, a previous study that assessed the metabolome of *S. passalidarum* in xylose fermentation encountered similar concentrations that were quantified here [33]. Possibly, as PYR is a branch-point metabolite involved in the respiration (mitochondria) and alcoholic fermentation (cytosol), this may have influenced the metabolomics analysis since the metabolomics is not able to separate the compounds from different compartments cellular [33]. Moreover, the precise quantification of such metabolites is limited due to its presence in more than one cellular compartment [35].

The changes in metabolite concentration do not readily allow conclusions on metabolic fluxes or the direction of the flux changes. An increase in metabolite concentration can both be indicative of the increased activity of metabolite producing enzymes, but also decreased the activity of metabolite consuming enzymes [36]. Nevertheless, the addition of the metabolite concentration in the MFA model can be useful to validated metabolome data. Here a correlation between carbon flux distribution measured and calculated could be done for 80% of studied metabolites. Thus, the stoichiometric network model and intracellular carbon fluxes distribution could be estimated more precisely. They were integrating MFA with metabolome data of xylose-fermenting yeasts. Therefore, demonstrating that metabolite concentration determines the intracellular metabolic flux distribution in the central metabolism of yeast [37].

### Intracellular carbon flux distribution of xylose metabolism in yeasts

The first step of xylose reduction to xylitol realized by xylose reductase (XR) enzyme using both NADPH and NADH as cofactors [1, 7, 8, 38]. However, the XR enzymes present different cofactor preference [7, 39–41]. Therefore, two reactions represent this first step in xylose metabolism in the model. First, the reaction is XYL + NADPH **→** XOL + NADP. Second reaction is XYL + NADH **→** XOL + NAD^+^. Xylose reduction reaction NADH-dependent showed approximately twice higher flux rate in *S. stipitis* (1.97) and *S. passalidarum* (1.28) that is observed in *S. arborariae* (0.68). In *S. arborariae,* the flux rate of conversion xylose to xylitol has employed the cofactor NADPH preferentially [7]. This feature observed in *S. arborariae* is associated with xylitol accumulation [7, 8]. Our results, corroborated with it and also demonstrated that only *S. arborariae* present xylitol production (Table 1). This characteristic may be associated with low xylose transportation capability [42]. The *S. arborariae* have a flux rate for oxidative-PPP (reaction G6P **→** RU5P) in 3.0 times higher than observed in *S. passalidarum*. This observation indicates the need to regenerate the NADPH cofactor in *S. arborariae*. Also, it noted that *S. arborariae* has a slower flux rate to consume xylose and a smaller growth rate. Due to the decreased cell growth, the requirement of NADPH has been reduced and caused the down-regulation of fluxes through the pentose phosphate pathway [13, 25].

On the contrary, for *S. passalidarum* already showed that it has two XR (genes XIL1.1 and XIL1.2), and one of them uses NADH preferentially as a cofactor [8]. Its enzymatic activity presents higher NADH-dependent XR [7]. Besides then, the conversion of G6P **→** RU5P in *S. passalidarum* was 3.0 times slower than *S. stipitis* and *S. arborariae.* Demonstrating less need for carbon flux to oxidative-PPP and carbon flux directed more to PPP and glycolysis pathways.

The enzymatic activities for xylose reductase (XR) of *S. stipitis* and *S. arborariae* cell extract are twice higher using NADPH as cofactor when compared to NADH-dependent activity [7, 8, 23]. Nevertheless, here, the calculated flux rates of the XR reaction using NADH was at least four times higher. Into MFA-measured was observed that the carbon flux distribution, preferably the use of NADH as the cofactor in XR reaction. This difference between enzymatic activities and calculated flux rates can be explained by optimal condition and concentration determined in enzymatic activities, not necessarily these occur in vivo. As observed previously, an MFA study showed the same result with flux distribution preferably using XR reaction with NAD-dependent in a recombinant xylose-utilizing *Saccharomyces cerevisiae* [23]. Therefore, the MFA showed that higher the reaction rate of XR NADH preference, more ethanol formation is observed (*S. stipitis* and *S. passalidarum*).

On the other hand, the XR NADPH preference showed xylitol accumulation (*S. arborariae*), confirming the literature data and our previous study [7, 8, 23]. The cofactor imbalance in XR and XDH reactions result in xylitol production, as observed in *S. arborariae* and prior studies [23]. The results of intracellular flux rates showed in MFA models are in good agreement with previous work that showed that higher NADH dependent XR activity resulted in less xylitol production [23].

Our results demonstrate a positive correlation between glycolytic flux rate and ethanol production. The low glycolytic flux seems to limit xylose utilization. These results are in agreement with a previous study that also applied metabolic flux analysis in genetic engineered *S. cerevisiae* [43, 44]. It has been previously suggested that the low glycolytic flux towards glyceraldehyde-3-phosphate and consequently, pyruvate may limit the consumption of xylose [43]. Therefore, increasing the metabolic reaction activities that direct carbon to glycolysis may be a valuable strategy to improve the metabolism of xylose. Take together, the metabolic flux along with the metabolome data, which increased the prediction accuracy, showed that in *S. stipitis* the glycolytic reaction (G6P **← →** F6P) is 4.5 times faster than *S. arborariae*, and 2.7 times faster than observed in *S. passalidarum*. The faster metabolic fluxes in the glycolytic pathway observed in *S. stipitis* resulted in better ethanol production, and it was the main characteristic that differentiated it from the other evaluated yeasts.

## Conclusions

The present study elucidated for the first time a stoichiometric model from xylose until ethanol to estimate the carbon flux distribution in *Spathaspora* yeasts for the first time. The metabolic flux model validated the quantification of 11 metabolites, where up to 80% of intracellular carbon flux rates could be correlated with an accuracy above 90%. The flux analysis corroborated that *S. stipitis* and *S. passalidarum* are the two yeasts with better metabolic characteristics towards xylose fermentation. These characteristics include higher xylose consumption rates, a higher reaction rate of XR with NADH preference, higher flux rates directed to PPP and glycolysis pathways, and less need to directed carbon flux to oxidative-PPP for the regeneration of NADPH. Characteristics that would enable better NADH/NAD^+^ balance, thus allowing improves ethanol production from xylose.

## Methods

### Yeast strains and cultivation conditions

The yeasts used in this study were *Scheffersomyces stipitis* (NRRL Y-7124), *Spathaspora arborariae* (NRRL Y-48658), and *Spathaspora passalidarum* (NRRL Y-27907). These were kindly provided by the ARS (NRRL) culture collection (Peoria, USA). All are preserved in 30% glycerol at − 80 °C. As described previously, were performed all cultivations in the bioreactors [7]. Briefly, the fermentations were carried out in bioreactors (Multifors 2, Infors HT) with 500 mL of the defined mineral medium [45], supplemented with 40 g L^− 1^ xylose as a carbon source. The fermentation started with an optical density of 600 nm (OD_600_) equals 0.5. The temperature set up at 28 °C and stirred was kept at 400 rpm, pH was maintained at 5.5 by addition 3 M KOH. Oxygen was supplied at limited conditions. At those conditions, dissolved oxygen (DO) was kept below 10% with airflow of only 0.05 L/min throughout the cultivation in the bioreactors. All fermentations were carried out in biological triplicates. Samples were withdrawn to determine xylose consumption and product formation at regular intervals of time (approximately every 8 h) of cultivation. Extra- and intracellular quantification of metabolites was done using samples in the middle exponential growth phase where a pseudo-steady state is assumed, and therefore, all rates at that time-points were considered as constant [15]. The time point of during exponential growth was 28 h, 32 h, and 40 h for *S. stipitis*, S*. arborariae*, and *S. passalidarum*, respectively.

### Determination of extracellular fluxes

The extracellular metabolites such as xylose, ethanol, xylitol, glycerol, acetate, pyruvate, and succinate concentrations were determined by High-Performance Liquid Chromatography (HPLC) as previously described [7]. Briefly, culture samples were collected in the middle exponential growth phase, centrifuged and the supernatant analyzed by an HPLC system (Acquity UPLC H Class, Waters) equipped with a refractive index detector. The metabolites were separated on an HPX-87 H column (Bio-Rad Laboratories) with a 5 mM sulfuric acid mobile phase at a flow rate of 0.6 mL/min and a temperature of 45 °C. After that, the extracellular concentration values are divided by biomass and time at that fermentation point. The data show the average ± standard deviation in mM/gCDW. h^− 1^ of three biological triplicates (Table 1). The carbon balance and degree of reduction were calculated by taking the ratio of products in C-mole and consumed substrate in C-mole [46]. Biomass was measured through OD_600_ using a spectrophotometer (SpectraMax M3, Molecular Devices). For each collected point, cell dry weight (CDW) was performed through 5 mL of pre-inoculum and stationary growth phase of all yeasts in fermentative processes. Samples were withdrawn and centrifuged (12,000×g, 5 min). Before weighing, the cells were placed in a glass tube and incubated to dry at 60 °C at least 48 h. Therefore, it established a correlation between OD_600_ values and CDW. Approximately, for each OD_600_ = 1.0 were obtained 0.5 g L^− 1^ of cells dry weight.

### Standard metabolites and solvents

Acetate (ACE), acetyl coenzyme A (ACCOA), alpha-ketoglutaric acid (AKG), dihydroxyacetone phosphate (DHAP), erythrose-4-phosphate (E4P), ethanol (EtOH), fructose-6-phosphate (F6P), glucose (GLU), glucose-6-phosphate (G6P), glyceraldehyde-3-phosphate (GAP), glycerol (GOL), malate (MAL), phosphoenolpyruvate (PEP), pyruvate (PYR), ribose-5-phosphate (R5P), ribulose-5-phosphate (RU5P), sedoheptulose-7-phosphate (S7P), succinate (SUC), xylitol (XOL), xylose (XYL), xylulose (XYLU) and all solvents as such as sulphuric acid, tributylamine, acetonitrile and methanol used in HPLC and UHPLC-MS/MS analysis were purchased from Sigma-Aldrich (St. Louis, MO, USA) in their highest purity. Ultrapure water (18.2 MΩ) was obtained from a Direct 16 Milli-Q purification system (Millipore, Bedford, USA).

### Metabolomics analysis

The experimental setup for determination and quantification of metabolome data is in Additional file 6. As mentioned before, all data was originated from the three replicates samples collected in the middle of the exponential growth phase under oxygen-limited conditions. This data point was the same used to calculate the extracellular flux rates, the carbon recovered, and redox balance. The sample preparation protocol and analytical data acquisition were previously described and optimized [26, 47, 48]. Briefly, preparation of the samples involved the steps of quenching and metabolites extraction using cold methanol (− 40 °C) followed by boiling ethanol (96 °C). The analytical method was based on UHPLC-MS/MS for metabolite separation and quantification [48]. Details of this analysis are previously showed [26, 48]. The MS methodology was carried out on an AcQuity^tm^ UPLC system (Waters, Milford, MA, USA) coupled to a triple quadrupole mass spectrometer (Xevo TQD, Waters) equipped with an electrospray ionization source. UPLC it performed on a Hydrophilic Interaction Liquid Chromatography (HILIC) with a BEH amide column (2.1 × 150 mm × 1.7 μm) (Waters Corporation, Milford, MA, USA) and Ion-Pairing Chromatography (IPC) with a reverse phase column, HSS-T3 (2.1 × 150 mm × 1.8 μm) (Waters Corporation, Milford, MA, USA). The metabolites detected in both environment extra and intracellular, for example, xylose, xylitol, and glycerol were not included into the metabolome dataset since the quantification of these compounds presents high variance, and it was not possible to define how much was intra- and extracellular.

### Statistical analysis of intracellular metabolite concentration

It performed in biological triplicates all cultivations in bioreactors. For each replicate, it extracted the intracellular metabolites in three-time points within the exponential growth phase of yeasts. The intracellular extraction of each time point was analyzed by UHPLC-MS/MS method in three technical replicates, giving a total of 27 samples measurements. Therefore, to compile all resulting data into a single value, the metabolome dataset was statistically processed through a measured repeated ANOVA design. RStudio software (http://www.rstudio.com, https://www.rstudio.com/products/rstudio/download/) was used to construct the ANOVA model. The following mathematical equation represents how the ANOVA model test was performed.

$$ y\_ ijk=\mu +\alpha \_i+\beta \_\left(j(i)\right)+\tau \_k+\left(\alpha \tau \right)\_ ik+e\_ ijk $$

y_ijk_ = μ + α_i_ + β_j(i)_ + τ_k_ + (ατ)_ik_ + e_ijk_Using this linear model, it was assumed that the data for class (*i)* for yeast (*j*) at time (*k*) is equal to an overall mean (μ) plus the treatment effect (αi), the effect of the yeast within that class (β_j(i)_) the effect of time (τk), the effect of the interaction between time and class ((ατ)_ik_) and the error (eijk).

Such that:

- μ = overall mean
- α_*i*_ = effect of class *i*
- β_*j(i)*_ = random effect of yeast *j* receiving class *i*
- τ_*k*_ = effect of time *k*
- (ατ)_*ik*_ = class by time interaction
- ε_*ijk*_ = experimental error

The Additional file 4 shows the concentrations obtained after statistical analysis from the metabolome data.

### Stoichiometric model construction

An overview of the metabolic model is shown in Additional file 1. The stoichiometric model was constructed based on previous studies [13, 23, 49]. The model composed of 39 reactions within the xylose assimilation pathway, pentose phosphate pathway, glycolysis, and TCA. It included the TCA cycle, but the compartmentalization into mitochondria and cytosol was not considered due to an equipment limitation. The probe of oxygen used covers only the measurements of dissolved oxygen in the medium. Thereby, it was not possible to measure the oxygen released, data necessary for the metabolic flux calculation. However, similar models have proved efficient to support understanding sugar metabolism in yeasts [23, 49]. Biomass equation was determined as previously described [23]. It consists of the macromolecules components of the cell (i.e., proteins, nucleic acid, and polysaccharides) [50]. The stoichiometric model was constructed based on the information available at The Kyoto Encyclopedia of Genes and Genomes (KEGG). It used as the reference genomic and biochemical information of *S. stipitis* (Entry T01023). The genes encoding for the enzymes on the carbohydrate metabolism present in the respective genome could be determined using KEGG pathway.

### Metabolic flux analysis using OptFlux

The model uploaded into OptFlux from an Excel file (Additional file 2). The degree of freedom of the metabolic network was calculated using the properties of the stoichiometric model. The accurate number of degrees of freedom obtained by the difference between the number of metabolites of the system and the number of linearly independent equations [17]. For differentiate internal and external reactions, external metabolites were identified with “[e]” and intracellular metabolites occurring in cytosolic subsystems with “[c].” It used a biomass reaction as an objective function [22, 23, 51]. Thus, added the extracellular measured flux rates obtained from of middle exponential growth phase to the model. The extracellular measurements used are xylose consumption rate, xylitol, acetate, glycerol, pyruvate, and succinate production rates. The simulations were run using the algebraic method with least square fitting as properties.

### Carbon flux distribution using extracellular flux rates measurements

The extracellular flux rates measurements were utilized to simulate the metabolic fluxes and to calculate the carbon fluxes distribution. The model classified as an overdetermined system containing 39 reactions, 35 metabolites, 27 genes, and 04 degrees of freedom. Nine extracellular flux rates were measured (xylose consumption, biomass, ethanol, carbon dioxide, xylitol, glycerol, acetate, pyruvate, and succinate productions) and used to limit the initial metabolic model resolution. Considering the principle of mass conservation and molarity, this ensures that the total amount of compounds produced must be equal to the total amount being consumed [17, 36, 52].

Between them, for each yeast, xylose consumption, and biomass production rates were maintained fixed during all simulations. Xylose consumption rates were fixed with following values − 2.15, − 0.90, and − 1.37 (mM/gCDW.h^− 1^), whereas biomass production rates were fixed with 1.45, 1.27, and 1.01 (g. L^− 1^) for *S. stipitis*, *S. arborariae,* and *S. passalidarum*, respectively. The extracellular ethanol flux rates were used to validate the carbon flux distribution in the calculated model. First, the simulations were performed without the ethanol flux rate, and then, the flux rate obtained through this simulation was compared with the ethanol rate obtained experimentally.

### Validation of metabolome data using metabolic flux analysis

The statistical analysis resulted in a single value of intracellular metabolites concentration. Those were added to the stoichiometric model. After that, a simulation was performed to determine the flux distribution within the metabolic network. Initially, the simulations performed with the addition of one metabolite by time. Thus, 11 measured fluxes distribution were obtained, one for each measured metabolite. Then, the fluxes derived from stoichiometric calculations and the ones with the addition of metabolome data were compared using the Pearson correlation coefficient (R^2^). The correlation coefficient is useful to find the relationship between the calculated and measured fluxes distributions. The metabolites, whose correlation was above 90%, were used in the stoichiometric model for a new round of carbon flux simulations. Thus it was possible to estimate the percentage of error, and consequently, identify hits between calculated and measured fluxes for each measured metabolite.

## Additional files

Additional file 1: Overview of metabolic network from xylose to ethanol. The metabolic model showns the directions of intracellular metabolic reactions (continuos arrows), xylose consumption and products formation (dashed arrows), and cofactors (NADPH/NADP^+^; NADH/NAD^+^; ATP) utilized in some reactions. (PDF 169 kb)

Additional file 2: The stoichiometric model. Metabolic reactions added to the OptFlux. (PDF 41 kb)

Additional file 3: List of metabolites. The intracellular and extracellular metabolites added to the OptFlux. (PDF 39 kb)

Additional file 4: Intracellular metabolites concentrations (μg/mL). Average and standart desviation of metabolites concentrations obtained after statistical analysis (ANOVA) from the metabolome data. (PDF 52 kb)

Additional file 5: **a** Correlation (R2) between calculated and measured fluxes - *S. stipitis*. Acetyl-CoA (ACCOA), dihydroxy-acetone-phosphate (DHAP), erythrose-4-phosphate (E4P), fructose-6-phosphate (F6P), glucose-6-phosphate (G6P), malate (MAL), phosphoenolpyruvate (PEP), pyruvate (PEP), ribose-5-phosphate (R5P), ribulose-5-phosphate (RU5P), and sedoheptulose-7-phosphate (S7P) were the metabolites measured. (X-axis) show the calculated fluxes using the constrained values of products formation. (Y-axis) show measured fluxes with respectively metabolites concentrations. Graphics in square presents a correlation higher than 0.9. Flux rates are in mmol/gCDW.h^− 1^. **b** Correlation (R2) between calculated and measured fluxes - *S. arborariae*. Acetyl-CoA (ACCOA), dihydroxy-acetone-phosphate (DHAP), erythrose-4-phosphate (E4P), fructose-6-phosphate (F6P), glucose-6-phosphate (G6P), malate (MAL), phosphoenolpyruvate (PEP), pyruvate (PYR), ribose-5-phosphate (R5P), ribulose-5-phosphate (RU5P), and sedoheptulose-7-phosphate (S7P) were the metabolites measured. (X-axis) show the calculated fluxes using the constrained values of products formation. (Y-axis) show measured fluxes with respectively metabolites concentrations. Graphics in square presents a correlation higher than 0.9. Flux rates are in mmol/gCDW.h^− 1^. **c** Correlation (R2) between calculated and measured fluxes - *S. passalidarum*. Acetyl-CoA (ACCOA), dihydroxy-acetone-phosphate (DHAP), erythrose-4-phosphate (E4P), fructose-6-phosphate (F6P), glucose-6-phosphate (G6P), malate (MAL), phosphoenolpyruvate (PEP), pyruvate (PYR), ribose-5-phosphate (R5P), ribulose-5-phosphate (RU5P), and sedoheptulose-7-phosphate (S7P) were the metabolites measured. (X-axis) show the calculated fluxes using the constrained values of products formation. (Y-axis) show measured fluxes with respectively metabolites concentrations. Graphics in square presents a correlation higher than 0.9. Flux rates are in mmol/gCDW.h^− 1^. (ZIP 183 kb)

Additional file 6: Experimental design for metabolomics data. Three species of xylose-fermenting yeasts *S. stipitis*, *S. arborariae*, and *S. passalidarum*. The times of replicates (T1, T2, T3) and the technical replicates (R1, R2, R3) are repeated for each biological replicate into an oxygen-limited condition (A, B, C). (PDF 135 kb)

## Data Availability

All data generated or analysed during this study are included in this published article and its supplementary information files.

## Abbreviations

ACCOA: Acetyl coenzyme A
ACE: Acetate
AKG: Alpha Ketoglutaric Acid
ANOVA: Analysis of variance
CDW: Cell dry weight
DHAP: Dihydroxy Acetone Phosphate
DR: Degree of reduction
E4P: Erythrose-4-phosphate
ETOH: Ethanol
F6P: D-Fructose-6-Phosphate
G6P: D-Glucose-6-Phosphate
GAP: Glyceraldehyde-3-phosphate
GLU: D-Glucose
GOL: Glycerol
HILIC: Hydrophilic Liquid Chromatography
HPLC: High-Performance Liquid Chromatography
IPC: Ion-Paring Chromatography
KEGG: Kyoto Encyclopedia of Genes and Genomes
KOH: Potassium Hydroxide
LC: Liquid Chromatography
MAL: Malate
MFA: Metabolic flux analysis
NAD^+^: Nicotinamide Adenine Dinucleotide
NADH: Nicotinamide Adenine Dinucleotide Reduced
NADP^+^: Nicotinamide Adenine Dinucleotide Phosphate
NADPH: Nicotinamide Adenine Dinucleotide Phosphate Reduced
OD_600_: Optical Density of 600 nm
PEP: Phosphoenolpyruvate
PPP: Pentose phosphate pathway
PYR: Pyruvate
R^2^: Person Correlation
R5P: D-Ribose-5-Phosphate
RU5P: D-Ribulose-5-Phosphate
S7P: Sedoheptulose-7-Phosphate
SUC: Succinate
TCA: Tricarboxylic acid
UHPLC-MS/MS: Ultra-High Performance Liquid Chromatography coupled to Tandem Mass Spectrometry
XDH: Xylitol Dehydrogenase
XOL: D-Xylitol
XR: Xylose reductase
XYL: D-Xylose
XYLU: D-Xylulose
