# Supplementary figures and images for: Metabolic flux analysis for metabolome data validation of naturally xylose-fermenting yeasts
Source: BMC Biotechnol. 2019 Aug 5;19:58. doi: 10.1186/s12896-019-0548-0 (PMC6683545; doi:10.1186/s12896-019-0548-0)

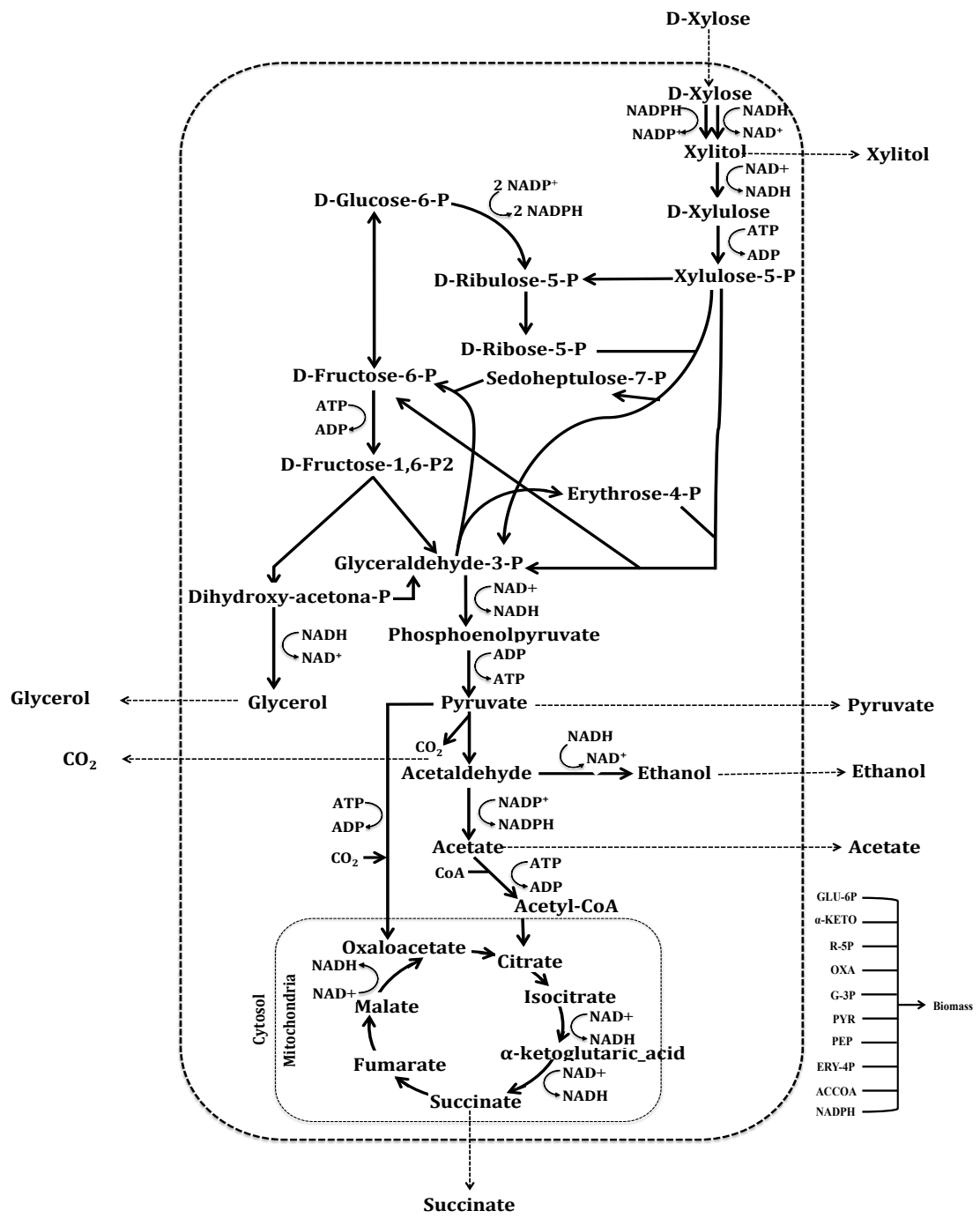

Supplement: Supplementary file 1 — Overview of metabolic network from xylose to ethanol. The metabolic model showns the directions of intracellular metabolic reactions (continuos arrows), xylose consumption and products formation (dashed arrows), and cofactors (NADPH/NADP+; NADH/NAD+; ATP) utilized in some reactions. (PDF 169 kb) [file 12896_2019_548_MOESM1_ESM.pdf]

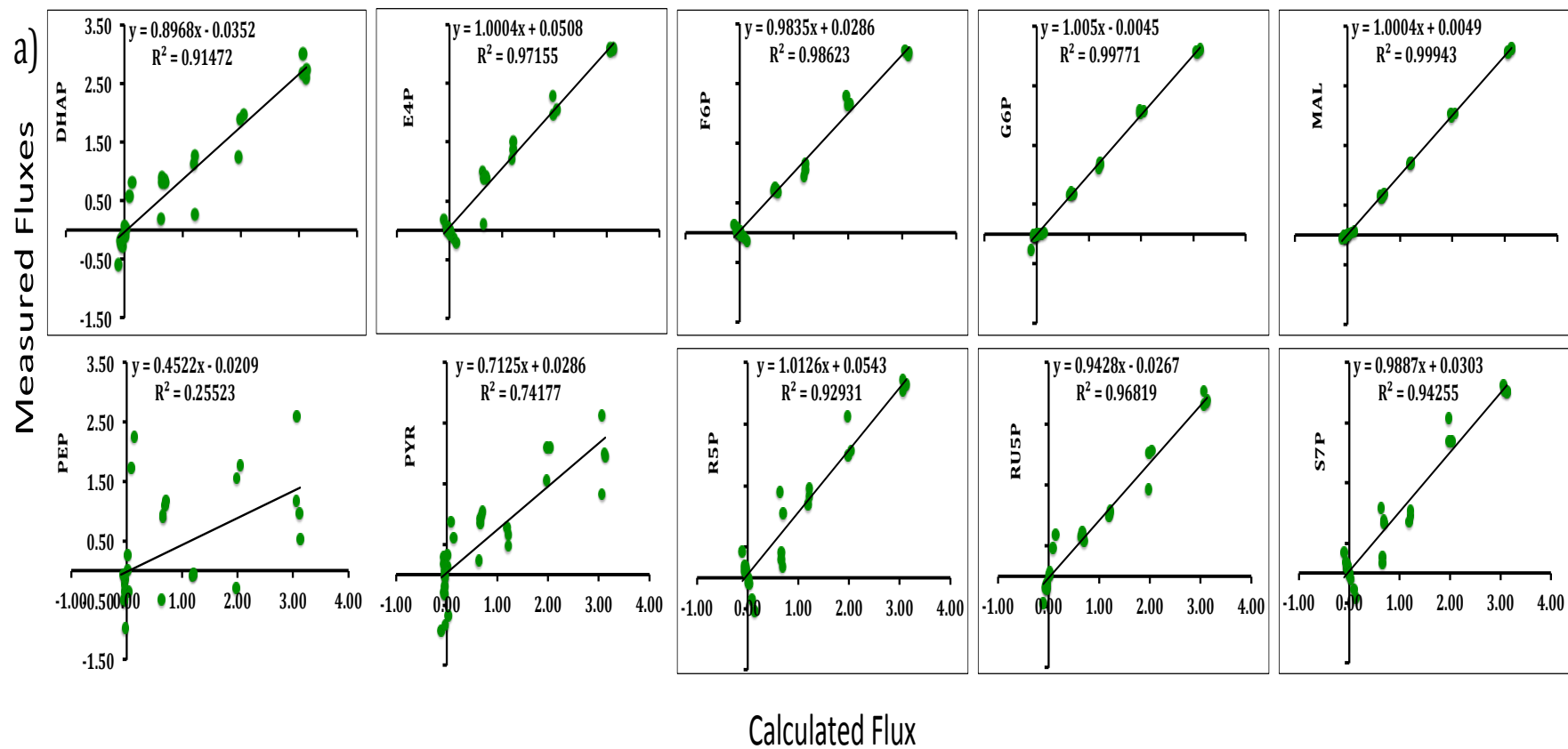

Supplement: Supplementary file 5 — a Correlation (R2) between calculated and measured fluxes - S. stipitis. Acetyl-CoA (ACCOA), dihydroxy-acetone-phosphate (DHAP), erythrose-4-phosphate (E4P), fructose-6-phosphate (F6P), glucose-6-phosphate (G6P), malate (MAL), phosphoenolpyruvate (PEP), pyruvate (PEP), ribose-5-phosphate (R5P), ribulose-5-phosphate (RU5P), and sedoheptulose-7-phosphate (S7P) were the metabolites measured. (X-axis) show the calculated fluxes using the constrained values of products formation. (Y-axis) show measured fluxes with respectively metabolites concentrations. Graphics in square presents a correlation higher than 0.9. Flux rates are in mmol/gCDW.h− 1. b Correlation (R2) between calculated and measured fluxes - S. arborariae. Acetyl-CoA (ACCOA), dihydroxy-acetone-phosphate (DHAP), erythrose-4-phosphate (E4P), fructose-6-phosphate (F6P), glucose-6-phosphate (G6P), malate (MAL), phosphoenolpyruvate (PEP), pyruvate (PYR), ribose-5-phosphate (R5P), ribulose-5-phosphate (RU5P), and sedoheptulose-7-phosphate (S7P) were the metabolites measured. (X-axis) show the calculated fluxes using the constrained values of products formation. (Y-axis) show measured fluxes with respectively metabolites concentrations. Graphics in square presents a correlation higher than 0.9. Flux rates are in mmol/gCDW.h− 1. c Correlation (R2) between calculated and measured fluxes - S. passalidarum. Acetyl-CoA (ACCOA), dihydroxy-acetone-phosphate (DHAP), erythrose-4-phosphate (E4P), fructose-6-phosphate (F6P), glucose-6-phosphate (G6P), malate (MAL), phosphoenolpyruvate (PEP), pyruvate (PYR), ribose-5-phosphate (R5P), ribulose-5-phosphate (RU5P), and sedoheptulose-7-phosphate (S7P) were the metabolites measured. (X-axis) show the calculated fluxes using the constrained values of products formation. (Y-axis) show measured fluxes with respectively metabolites concentrations. Graphics in square presents a correlation higher than 0.9. Flux rates are in mmol/gCDW.h− 1. (ZIP 183 kb) [file 12896_2019_548_MOESM5_ESM.zip › Supplementary file 5a.pdf]

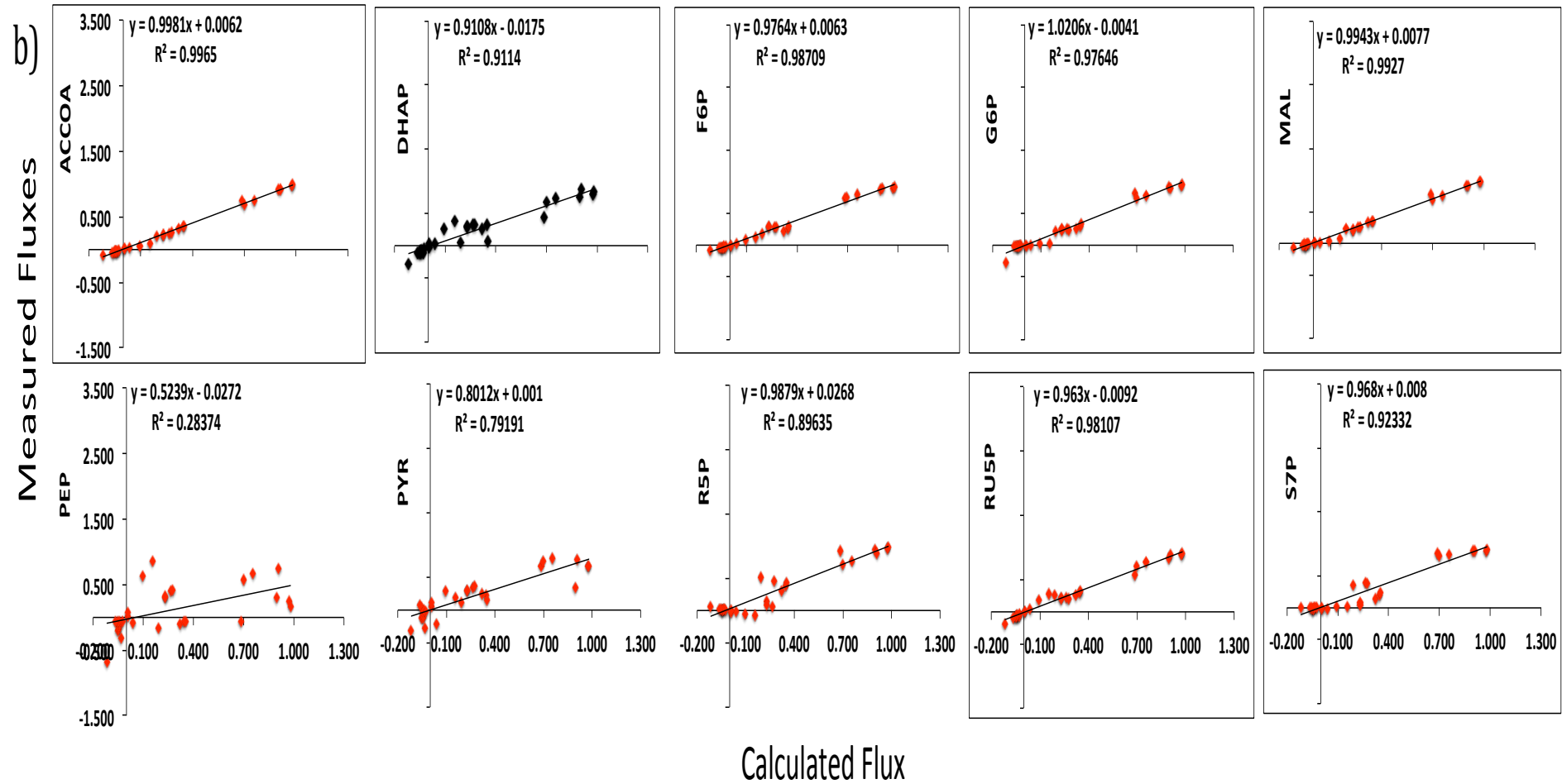

Supplement: Supplementary file 5 — a Correlation (R2) between calculated and measured fluxes - S. stipitis. Acetyl-CoA (ACCOA), dihydroxy-acetone-phosphate (DHAP), erythrose-4-phosphate (E4P), fructose-6-phosphate (F6P), glucose-6-phosphate (G6P), malate (MAL), phosphoenolpyruvate (PEP), pyruvate (PEP), ribose-5-phosphate (R5P), ribulose-5-phosphate (RU5P), and sedoheptulose-7-phosphate (S7P) were the metabolites measured. (X-axis) show the calculated fluxes using the constrained values of products formation. (Y-axis) show measured fluxes with respectively metabolites concentrations. Graphics in square presents a correlation higher than 0.9. Flux rates are in mmol/gCDW.h− 1. b Correlation (R2) between calculated and measured fluxes - S. arborariae. Acetyl-CoA (ACCOA), dihydroxy-acetone-phosphate (DHAP), erythrose-4-phosphate (E4P), fructose-6-phosphate (F6P), glucose-6-phosphate (G6P), malate (MAL), phosphoenolpyruvate (PEP), pyruvate (PYR), ribose-5-phosphate (R5P), ribulose-5-phosphate (RU5P), and sedoheptulose-7-phosphate (S7P) were the metabolites measured. (X-axis) show the calculated fluxes using the constrained values of products formation. (Y-axis) show measured fluxes with respectively metabolites concentrations. Graphics in square presents a correlation higher than 0.9. Flux rates are in mmol/gCDW.h− 1. c Correlation (R2) between calculated and measured fluxes - S. passalidarum. Acetyl-CoA (ACCOA), dihydroxy-acetone-phosphate (DHAP), erythrose-4-phosphate (E4P), fructose-6-phosphate (F6P), glucose-6-phosphate (G6P), malate (MAL), phosphoenolpyruvate (PEP), pyruvate (PYR), ribose-5-phosphate (R5P), ribulose-5-phosphate (RU5P), and sedoheptulose-7-phosphate (S7P) were the metabolites measured. (X-axis) show the calculated fluxes using the constrained values of products formation. (Y-axis) show measured fluxes with respectively metabolites concentrations. Graphics in square presents a correlation higher than 0.9. Flux rates are in mmol/gCDW.h− 1. (ZIP 183 kb) [file 12896_2019_548_MOESM5_ESM.zip › Supplementary file 5b.pdf]

c)

Measured Fluxes

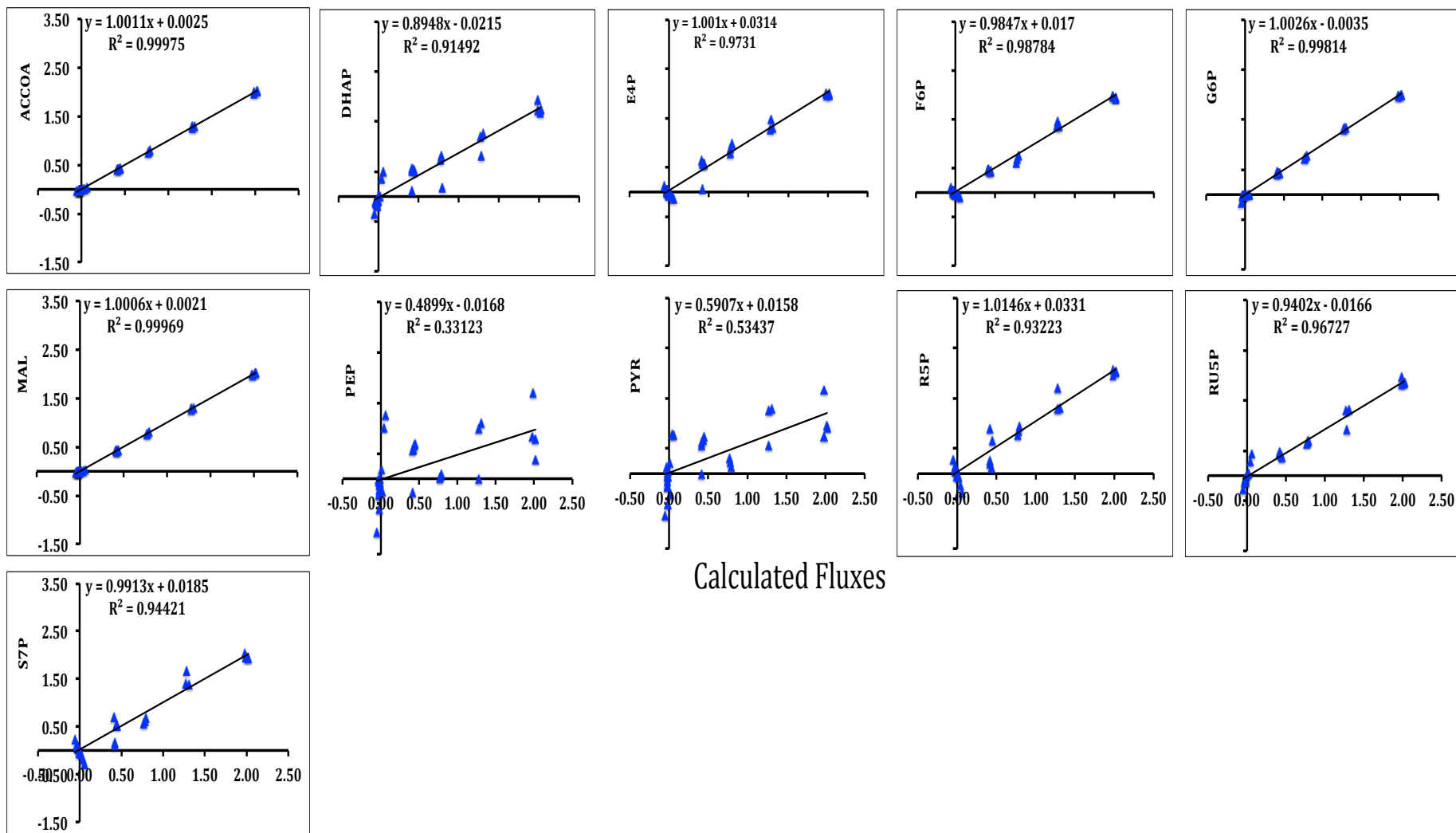

Supplement: Supplementary file 5 — a Correlation (R2) between calculated and measured fluxes - S. stipitis. Acetyl-CoA (ACCOA), dihydroxy-acetone-phosphate (DHAP), erythrose-4-phosphate (E4P), fructose-6-phosphate (F6P), glucose-6-phosphate (G6P), malate (MAL), phosphoenolpyruvate (PEP), pyruvate (PEP), ribose-5-phosphate (R5P), ribulose-5-phosphate (RU5P), and sedoheptulose-7-phosphate (S7P) were the metabolites measured. (X-axis) show the calculated fluxes using the constrained values of products formation. (Y-axis) show measured fluxes with respectively metabolites concentrations. Graphics in square presents a correlation higher than 0.9. Flux rates are in mmol/gCDW.h− 1. b Correlation (R2) between calculated and measured fluxes - S. arborariae. Acetyl-CoA (ACCOA), dihydroxy-acetone-phosphate (DHAP), erythrose-4-phosphate (E4P), fructose-6-phosphate (F6P), glucose-6-phosphate (G6P), malate (MAL), phosphoenolpyruvate (PEP), pyruvate (PYR), ribose-5-phosphate (R5P), ribulose-5-phosphate (RU5P), and sedoheptulose-7-phosphate (S7P) were the metabolites measured. (X-axis) show the calculated fluxes using the constrained values of products formation. (Y-axis) show measured fluxes with respectively metabolites concentrations. Graphics in square presents a correlation higher than 0.9. Flux rates are in mmol/gCDW.h− 1. c Correlation (R2) between calculated and measured fluxes - S. passalidarum. Acetyl-CoA (ACCOA), dihydroxy-acetone-phosphate (DHAP), erythrose-4-phosphate (E4P), fructose-6-phosphate (F6P), glucose-6-phosphate (G6P), malate (MAL), phosphoenolpyruvate (PEP), pyruvate (PYR), ribose-5-phosphate (R5P), ribulose-5-phosphate (RU5P), and sedoheptulose-7-phosphate (S7P) were the metabolites measured. (X-axis) show the calculated fluxes using the constrained values of products formation. (Y-axis) show measured fluxes with respectively metabolites concentrations. Graphics in square presents a correlation higher than 0.9. Flux rates are in mmol/gCDW.h− 1. (ZIP 183 kb) [file 12896_2019_548_MOESM5_ESM.zip › Supplementary file 5c.pdf]

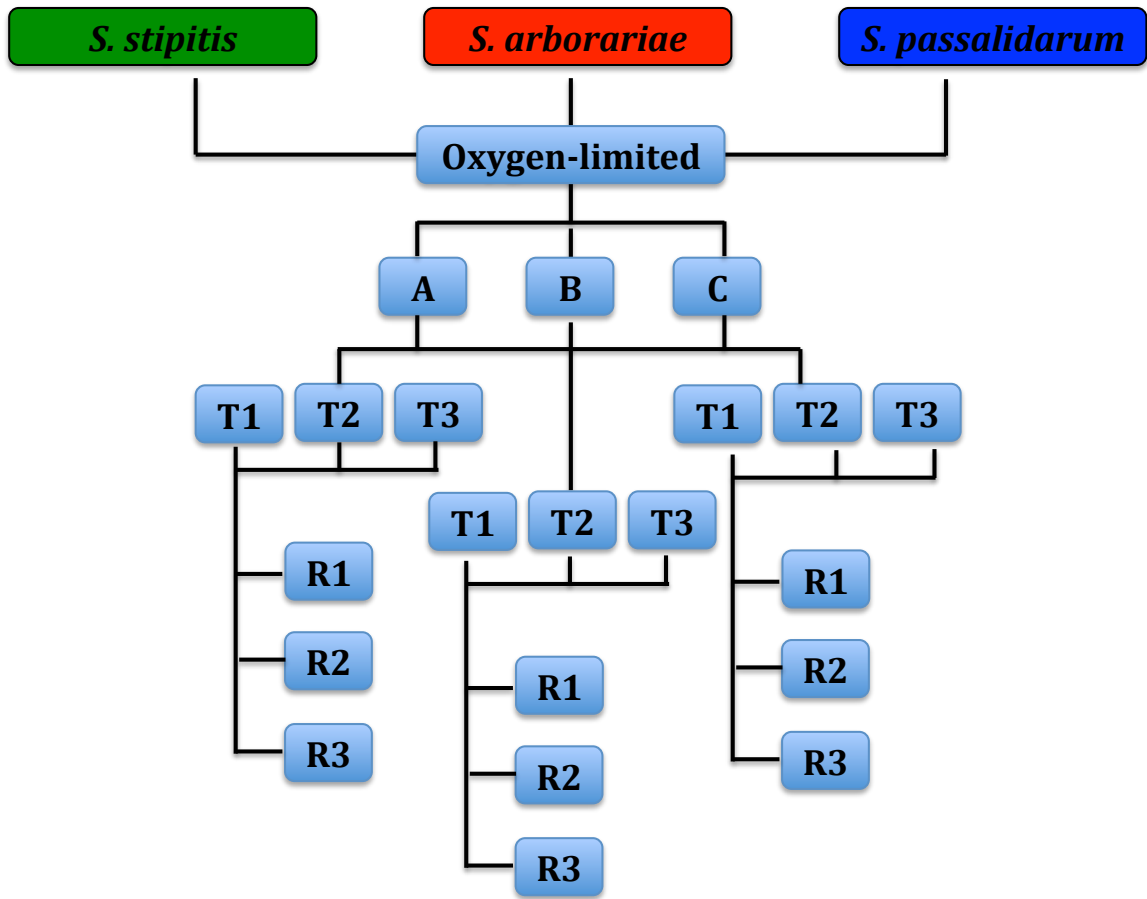

Supplement: Supplementary file 6 — Experimental design for metabolomics data. Three species of xylose-fermenting yeasts S. stipitis, S. arborariae, and S. passalidarum. The times of replicates (T1, T2, T3) and the technical replicates (R1, R2, R3) are repeated for each biological replicate into an oxygen-limited condition (A, B, C). (PDF 135 kb) [file 12896_2019_548_MOESM6_ESM.pdf]
