# Supplementary material for: Metabolic flux analysis for metabolome data validation of naturally xylose-fermenting yeasts
Source: BMC Biotechnol. 2019 Aug 5;19:58. doi: 10.1186/s12896-019-0548-0 (PMC6683545; doi:10.1186/s12896-019-0548-0)
Supplement: Supplementary file 2 — The stoichiometric model. Metabolic reactions added to the OptFlux. (PDF 41 kb) [file 12896_2019_548_MOESM2_ESM.pdf]

ID	Reactions equation	EC number	Pathways
R01	D-Xylose[c] + NADPH[c] --> Xylitol[c] + NADP[c]	1.1.1.307	Xylose Assimilation Pathway
R02	D-Xylose[c] + NADH[c] --> Xylitol[c] + NAD[c]	1.1.1.307	Xylose Assimilation Pathway
R03	Xylitol[c] + NAD[c] --> D-Xylulose[c] + NADH[c]	1.1.1.9	Xylose Assimilation Pathway
R04	D-Xylulose[c] + ATP[c] --> D-Xylulose_5-phosphate[c] + ADP[c]	2.7.1.17	Xylose Assimilation Pathway
R05	D-Xylulose_5-phosphate[c] --> D-Ribulose_5-phosphate[c]	5.1.3.1	Pentose Phosphate Pathway
R06	D-Xylulose_5-phosphate[c] + D-Erythrose_4-phosphate[c] --> D-Fructose_6-phosphate[c] + D-Glyceraldehyde_3-phosphate[c]	2.2.1.1	Pentose Phosphate Pathway
R07	D-Xylulose_5-phosphate[c] + D-Ribose_5-phosphate[c] --> Sedoheptulose_7-phosphate[c] + D-Glyceraldehyde_3-phosphate[c]	2.2.1.1	Pentose Phosphate Pathway
R08	D-Ribulose_5-phosphate[c] --> D-Ribose_5-phosphate[c]	5.3.1.6	Pentose Phosphate Pathway
R09	Sedoheptulose_7-phosphate[c] + D-Glyceraldehyde_3-phosphate[c] --> D-Erythrose_4-phosphate[c] + D-Fructose_6-phosphate[c]	2.2.1.2	Pentose Phosphate Pathway
R10	D-Glucose_6-phosphate[c] + 2 NADP[c] --> D-Ribulose_5-phosphate[c] + CO2[c] + 2 NADPH[c]	1.1.1.49	Pentose Phosphate Pathway
R11	D-Glucose_6-phosphate[c] <--> D-Fructose_6-phosphate[c]	5.3.1.9	Glycolisis Pathway
R12	D-Fructose_6-phosphate[c] + ATP[c] <--> D-fructose-1-6-bisphosphate[c] + ADP[c]	2.7.1.11	Glycolisis Pathway
R13	D-fructose-1-6-bisphosphate[c] --> Dihydroxyacetone_phosphate[c] + D-Glyceraldehyde_3-phosphate[c]	4.1.2.13	Glycolisis Pathway
R14	Dihydroxyacetone_phosphate[c] --> D-Glyceraldehyde_3-phosphate[c]	5.3.1.1	Glycolisis Pathway
R15	Dihydroxyacetone_phosphate[c] + NADH[c] --> Glycerol[c] + NAD[c]	1.1.1.156	Glycolisis Pathway
R16	D-Glyceraldehyde_3-phosphate[c] + NAD[c] --> Phosphoenolpyruvate[c] + NADH[c]	1.2.1.12	Glycolisis Pathway
R17	Phosphoenolpyruvate[c] + ADP[c] --> Pyruvate[c] + ATP[c]	2.7.1.40	Pyruvaye Metabolism
R18	Pyruvate[c] --> Acetaldehyde[c] + CO2[c]	4.1.1.1	Pyruvaye Metabolism
R19	Acetaldehyde[c] + NADH[c] --> Ethanol[c] + NAD[c]	1.1.1.1	Pyruvaye Metabolism
R20	Acetaldehyde[c] + NADP[c] --> Acetate[c] + NADPH[c]	1.2.1.5	Pyruvaye Metabolism
R21	Acetate[c] + CoA[c] + ATP[c] --> Acetyl_CoA[c] + ADP[c] + Pi[c]	6.2.1.1	Pyruvaye Metabolism
R22	Pyruvate[c] + CO2[c] + ATP[c] --> Oxaloacetate[c] + ADP[c] + Pi[c]	6.4.1.1	TCA Cicle Pathway
R23	Acetyl_CoA[c] + Oxaloacetate[c] --> Citrate[c] + CoA[c]	2.3.3.1	TCA Cicle Pathway
R24	Citrate[c] --> Isocitrate[c]	4.2.1.3	TCA Cicle Pathway
R25	Isocitrate[c] + NAD[c] --> alpha-Ketoglutaric_acid[c] + CO2[c] + NADH[c]	1.1.1.41	TCA Cicle Pathway
R26	alpha-Ketoglutaric_acid[c] + NAD[c] --> Succinate[c] + NADH[c] + CO2	1.2.4.2	TCA Cicle Pathway
R27	Succinate[c] --> Fumarate[c]	1.3.5.1	TCA Cicle Pathway
R28	Fumarate[c] --> Malate[c]	4.2.1.2	TCA Cicle Pathway
R29	Malate[c] + NAD[c] --> Oxaloacetate[c] + NADH[c]	1.1.1.37	TCA Cicle Pathway
R30	D-Xylose[e] --> D-Xylose[c]		External Metabolite
R31	Xylitol[c] --> Xylitol[e]		External Metabolite
R32	Glycerol[c] --> Glycerol[e]		External Metabolite
R33	CO2[c] --> CO2[e]		External Metabolite
R34	Ethanol[c] --> Ethanol[e]		External Metabolite
R35	Pyruvate[c] --> Pyruvate[e]		External Metabolite
R36	Acetate[c] --> Acetate[e]		External Metabolite
R37	Succinate[c] --> Succinate[e]		External Metabolite
R38	0.300 alpha-Ketoglutaric_acid[c] + 0.287 Oxaloacetate[c] + 0.406 Pyruvate[c] + 0.061 D-Erythrose_4-phosphate[c] +<br>0.040 D-Ribose_5-phosphate[c] + 0.164 D-Glyceraldehyde_3-phosphote[c] + 0.122 Phosphoenolpyruvate[c] +<br>0.176 Acetyl_CoA[c] + 1.762 NADPH[c] --> 0.071 Fumarate[c] + 0.026 Acetate[c] + 0.335 NADH[c] + 1.521 Pi[c] + 0.323 CO2[c]		Biomass_reaction
R39	Biomass[c] --> Biomass_formation[e]		Biomass_formation
