# Supplementary material for: Metabolic flux analysis for metabolome data validation of naturally xylose-fermenting yeasts
Source: BMC Biotechnol. 2019 Aug 5;19:58. doi: 10.1186/s12896-019-0548-0 (PMC6683545; doi:10.1186/s12896-019-0548-0)
Supplement: Supplementary file 3 — List of metabolites. The intracellular and extracellular metabolites added to the OptFlux. (PDF 39 kb) [file 12896_2019_548_MOESM3_ESM.pdf]

ID	Metabolites	Formula	Compartment
XYL[c]	D-Xylose[c]	C5H10O5	cytosol
XOL[c]	Xylitol[c]	C5H12O5	cytosol
XYLU[c]	D-Xylulose[c]	C5H10O5	cytosol
XYLU-5P[c]	D-Xylulose_5-phosphate[c]	C5H11O8P	cytosol
ERY-4P[c]	D-Erythrose_4-phosphate[c]	C4H9O7P	cytosol
FRU-6P[c]	D-Fructose_6-phosphate[c]	C6H13O9P	cytosol
F1-6-BP[c]	D-fructose-1-6-bisphosphate[c]	C6H14O12P2	cytosol
GA-3P[c]	D-Glyceraldehyde_3-phosphate[c]	C3H7O6P	cytosol
RIB-5P[c]	D-Ribose_5-phosphate [c]	C5H11O8P	cytosol
SEDO-7P[c]	Sedoheptulose_7-phosphate[c]	C7H15O10P	cytosol
RIBU-5P[c]	D-Ribulose_5-phosphate[c]	C5H11O8P	cytosol
GLU-6P[c]	D-Glucose_6-phosphate[c]	C6H13O9P	cytosol
DHAP[c]	Dihydroxyacetone_phosphate[c]	C3H7O6P	cytosol
3PGT[c]	3-Phosphoglycerate[c]	C3H7O7P	cytosol
GROL[c]	Glycerol[c]	C3H8O3	cytosol
GLY-3P[c]	Glycerol_3-phosphate[c]	C3H9O6P	cytosol
PEP[c]	Phosphoenolpyruvate[c]	C3H5O6P	cytosol
PYR[c]	Pyruvate[c]	C3H4O3	cytosol
ACDH[c]	Acetaldehyde[c]	C2H4O	cytosol
ETOH[c]	Ethanol[c]	C2H6O	cytosol
ACE[c]	Acetate[c]	C2H4O2	cytosol
ACCOA[c]	Acetyl-CoA[c]	C23H38N7O17P3S	cytosol
COA[c]	CoA[c]	C21H36N7O16P3S	cytosol
OXA[c]	Oxaloacetate[c]	C4H4O5	cytosol
CIT[c]	Citrate[c]	C6H8O7	cytosol
ISO[c]	Isocitrate[c]	C6H8O7	cytosol
AKG[c]	alpha-Ketoglutaric_acid[c]	C5H6O5	cytosol
FUM[c]	Fumarate[c]	C4H4O4	cytosol
SUC[c]	Succinate[c]	C4H6O4	cytosol
MAL[c]	Malate[c]	C4H6O5	cytosol
CO2[c]	Carbon_dioxide[c]	CO2	cytosol
Pi[c]	Phosphate[c]	H3PO4	cytosol
H2O[c]	H2O[c]	H2O	cytosol
NADPH[c]	NADPH[c]	C21H30N7O17P3	cytosol
NADP[c]	NADP[c]	C21H29N7O17P3	cytosol
NADH[c]	NADH[c]	C21H29N7O14P2	cytosol
NAD[c]	NAD[c]	C21H28N7O14P2	cytosol
ATP[c]	ATP[c]	C10H16N5O13P3	cytosol
ADP[c]	ADP[c]	C10H15N5O10P2	cytosol
Biomass[c]	Biomass[c]	CH1.8O0.5N0.2	cytosol
XYL[e]	D-Xylose[e]	C5H10O5	external
XOL[e]	Xylitol[e]	C5H12O5	external
GROL[e]	Glycerol[e]	C3H8O3	external
ETOH[e]	Ethanol[e]	C2H6O	external
PYR[e]	Pyruvate[e]	C3H4O3	external
ACE[e]	Acetate[e]	C2H4O2	external
SUC[e]	Succinate[e]	C4H6O4	external
CO2[e]	Carbon_dioxide[e]	CO2	external
