# Supplementary material for: Metabolic flux analysis for metabolome data validation of naturally xylose-fermenting yeasts
Source: BMC Biotechnol. 2019 Aug 5;19:58. doi: 10.1186/s12896-019-0548-0 (PMC6683545; doi:10.1186/s12896-019-0548-0)
Supplement: Supplementary file 4 — Intracellular metabolites concentrations (μg/mL). Average and standart desviation of metabolites concentrations obtained after statistical analysis (ANOVA) from the metabolome data. (PDF 52 kb) [file 12896_2019_548_MOESM4_ESM.pdf]

METABOLITES		*S. stipitis* (28h)		*S. arborariae* (32h)		*S. passalidarum* (45h)	
		**µg/mL**	**s**	**µg/mL**	**s**	**µg/mL**	**s**
**1.**	ACCOA	-	-	8.58	2.07	10.12	0.04
**2.**	AKG	-	-	-	-	-	-
**3.**	MAL	28.09	2.88	15.74	1.92	56.33	0.55
**4.**	G6P	6.24	1.38	4.59	1.94	16.60	2.18
**5.**	F6P	5.13	1.46	8.55	1.03	21.10	0.89
**6.**	DHAP	3.50	0.30	0.88	0.32	2.33	0.01
**7.**	GAP	-	-	-	-	-	-
**8.**	R5P	4.19	0.52	2.21	0.02	6.69	0.52
**9.**	Ru5P	11.81	1.66	8.15	2.10	11.93	0.81
**10.**	E4P	1.89	0.14	-	-	2.66	0.02
**11.**	S7P	19.94	3.91	17.39	2.73	140.06	11.85
**12.**	PEP	3.53	0.42	4.73	0.82	13.82	0.39
**13.**	PYR	8.61	0.28	4.73	0.78	5.09	0.83
**14.**	Xylu	-	-	-	-	-	-
**15.**	Xylose	3389.29	841.20	5116.46	312.05	1632.66	504.82
**16.**	Glucose	-	-	-	-	-	-
**17.**	Glycerol	-	-	-	-	-	-
**18.**	Xylitol	221.58	79.43	422.62	29.84	321.48	132.57
